# Genome-wide methylation analysis identified sexually dimorphic methylated regions in hybrid tilapia

**DOI:** 10.1038/srep35903

**Published:** 2016-10-26

**Authors:** Zi Yi Wan, Jun Hong Xia, Grace Lin, Le Wang, Valerie C. L. Lin, Gen Hua Yue

**Affiliations:** 1 Temasek Life Sciences Laboratory, National University of Singapore, 1 Research Link, 117604 Singapore; 2 School of Biological Sciences, Nanyang Technological University, 6 Nanyang Drive, 637551 Singapore; 3 State Key Laboratory of Biocontrol, Institute of Aquatic Economic Animals and Guangdong Provincial Key Laboratory for Aquatic Economic Animals, College of Life Sciences, Sun Yat-Sen University, Guangzhou 510275, PR China; 4 Department of Biological Sciences, National University of Singapore, 14 Science Drive, 117543 Singapore

## Abstract

Sexual dimorphism is an interesting biological phenomenon. Previous studies showed that DNA methylation might play a role in sexual dimorphism. However, the overall picture of the genome-wide methylation landscape in sexually dimorphic species remains unclear. We analyzed the DNA methylation landscape and transcriptome in hybrid tilapia (*Oreochromis spp.*) using whole genome bisulfite sequencing (WGBS) and RNA-sequencing (RNA-seq). We found 4,757 sexually dimorphic differentially methylated regions (DMRs), with significant clusters of DMRs located on chromosomal regions associated with sex determination. CpG methylation in promoter regions was negatively correlated with the gene expression level. MAPK/ERK pathway was upregulated in male tilapia. We also inferred active cis-regulatory regions (ACRs) in skeletal muscle tissues from WGBS datasets, revealing sexually dimorphic cis-regulatory regions. These results suggest that DNA methylation contribute to sex-specific phenotypes and serve as resources for further investigation to analyze the functions of these regions and their contributions towards sexual dimorphisms.

Sexual dimorphism (SD) is a common phenomenon and refers to differences of features that discriminate between males and females. These features are very diverse, ranging from external (e.g. color, shape, size, and structure) to internal (e.g. gene expressions). They are often linked with reproduction. Previous studies on SD focused on examining the relationships among sexual dimorphism, sexual selection and reproduction^1^. Phenotypes are determined by genetics, epigenetics, environmental factors and their interactions. In molecular genetics, gene regulation involves the spatial and temporal modulation of gene transcription^2^. One such genetic regulatory network is CpG methylation in the gene promoter region, which silences the corresponding gene. The addition of a methyl group to the fifth carbon of a cytosine base (5 mC) and adenine methylation is the major form of DNA methylation, with CpG methylation being the most studied form^3^. Other forms of cytosine methylations are CHH and CHG, with H representing adenine (A), guanine (G) or thymine (T)^4^,^5^. In animals, cytosine methylation almost consists exclusively of CpG methylation, although there are low levels of CHH and CHG methylation found in embryonic stem cells^6^. These stable epigenetic markers are heavily involved in many cellular functions including cell differentiation, gene regulation, suppressing transposable elements, germ cell formation, genomic imprinting and X-chromosome inactivation^5^,^7^. There are evidences that gene regulation has a major role in creating sex-specific differences^1^,^8^,^9^,^10^,^11^ For example, in *Drosophila melanogaster*, sexually dimorphic abdominal pigmentation is caused by differences in gene regulation between males and females^12^. One example of gene regulation is DNA methylation on CpG islands located on the promoter region of the targeted gene. Recent studies showed that epigenetic factors such as DNA methylation may play an important role in SD^13^. Recent developments in CpG methylation analysis have shed light on the function and information content of DNA methylation in terms of cis-regulatory functions^14^,^15^,^16^. Reports have shown that DNA methylation marks and transcription factor binding motifs can serve as a highly informative epigenetic marker to map cis-regulatory elements in a given genome^16^. These cis-regulatory elements include well-studied elements such as enhancers and promoters^14^, which are responsible for regulating development and physiology by acting as a control switch for gene expression^14^,^15^. However, our understanding of the molecular mechanisms underlying sex dimorphisms is still very limited.

In teleost fish, SD varies significantly between species. In zebrafish and medaka, two popular model species in developmental biology, no significant sexual size dimorphism (SSD) is observed^11^. In half-smooth tongue sole (*Cynoglossus semilaevis*), a female-biased SSD is observed, with females achieving body-sizes 2–4 times of that of males^13^. In *Lamprologous callipterus*, an extreme male-biased SSD takes place^17^. However, the molecular mechanisms responsible for many sexually dimorphic traits in teleosts have remained mostly undetermined. Nile tilapia (*Oreochromis niloticus*) and Mozambique tilapia (*Oreochromis mossambicus*) belong to the *Oreochromini* monophyletic group and are the second most important group of aquaculture species in the world^18^. They have an important evolutionary position, being the basal species to the East African radiations of cichlid fishes^18^. These species exhibit a male-biased sexual size dimorphism, in which males have a higher growth rate compared to females in both species. The superior growth rate of male tilapias has driven large sectors of the tilapia aquaculture industry to develop all-male tilapia populations for production using sex-reversal techniques^18^. In addition, the genome sequence of a Nile tilapia has been sequenced^19^, and other genomic resources, including microsatellites^20^, genome-wide SNPs^21^,^22^, linkage maps^23^,^24^,^25^ and QTL for important traits^25^,^26^, are available. Hence, tilapia is a good model for studying the molecular mechanisms underlying SD. In fish, a few studies on the relationship between sex and DNA methylation were conducted. For example, in European sea bass, an increase in CpG methylation in the promoter of cyp19a1a suppressed *cyp19a1a* expression and female gonad development^27^. In *Cynoglossus semilaevis*, genome-wide analysis of DNA methylation revealed epigenetic modification and inheritance in sexual reversal of fish^13^. However, systematic searches for sex-differences in genome-wide DNA methylation have not been conducted in tilapia. Therefore, we undertook a genome-wide search for sexually dimorphic genetic and epigenetic effects in tilapia leading to SSD to shed some new lights on mechanisms underlying SSD.

In this study, we applied RNA-seq and whole genome bisulfite sequencing (WGBS) on hybrid tilapia (*O. mossambicus* X *O. niloticus*) skeletal muscle tissue to investigate the gene expression and cytosine methylation landscape on a genome-wide scale to understand the molecular mechanisms of SSDs. Muscle growth is an important economic trait in tilapia aquaculture. The weight of tilapia fillet contributes heavily towards the yield per fish, which is an important parameter in the aquaculture industry^18^,^28^. Here, we report the first methylome of skeletal muscle tissue, including sexually dimorphic DMRs, in hybrid tilapia, which is genetically close to Nile tilapia^21^,^22^. We discovered that MAPK/ERK signaling pathway overexpression in male tilapia was associated with superior growth rate in male tilapia. We inferred and mapped putative active cis-regulatory regions in tilapia. This study provides the first genome wide molecular analysis of tilapia SSD while the cis-regulatory regions mapped will serve as important genetic toolkits for tilapia gene regulatory analysis.

## Results and Discussion

### Single base-pair resolution methylome of hybrid tilapia

Skeletal muscles from two male and two female hybrid tilapias (*O. niloticus* X *O. mossambicus*) have been sampled for this study. High molecular weight DNA extracted from each tilapia individual was spiked with unmethylated lambda DNA (Promega, Fitchburg, USA) and subjected to bisulfite conversion before library construction, and sequenced on an Illumina HiSeq 2000. A total of 107.6 Gb of sequencing data averaging 26.69 Gb per sample were obtained for the whole genome bisulfite sequencing of tilapia muscle tissue. A statistical summary of various sequencing result parameters is reported in Supplementary Table 1. Clean reads filtered using NGS QC Toolkits^29^ sum up to a total of 242 million read pairs, averaging ~60 million read pairs per library. An average of 182,639 reads were mapped onto the Lambda DNA reference genome per library, out of which, on average, 0.30% of the cytosines were read as C instead of T, indicating that the bisulphite conversion efficiency is 99.7%. Reads were then mapped to the oreNil2 Nile tilapia reference genome assembly using a three-letter aligner, *bismark*^30^. We used bowtie2 as bowtie2 gives better performance for reads with read length longer than 50 bp^31^. We allowed one mismatch per read to increase the sensitivity of our mapping process, although this came at a cost of higher computing power requirement and a longer mapping time.

**Figure 1:**
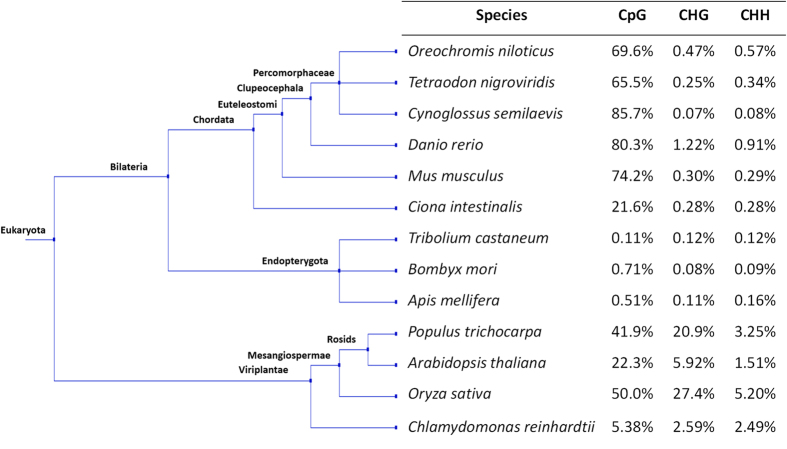
Overall methylation levels in 13 species of eukaryotes, including *Oreochromis niloticus*. Tree topology is generated from NCBI Taxonomy. The DNA methylation levels are from the main chromosomes of each organism, whereby chloroplast and mitochondria genomes are not included. Cytosine methylation data for *T. nigroviridis*, *C. intestinalis*, *T. castaneum*, *B. mori*, *A. mellifera* and *O. sativa* are taken from Zemach *et al*.^34^. Cytosine methylation data for *D. rerio*, *M. musculus*, *P. trichocarpa*, *A. thaliana*, *C. reinhardtii* are taken from Feng *et al*.^36^. Cytosine methylation data for *C. semilaevis* are taken from Shao *et al*.^13^.

The mapping efficiency for each library is 56.1%, 56.7%, 54.6% and 54.0% for Male-1 (M1), Male-2 (M2), Female-1 (F1) and Female-2 (F2) libraries, respectively. The mapping efficiency of whole genome bisulphite sequencing on a well-constructed genome such as the human genome (hg18) was reported to be 64.20% using the same parameters as ours for bismark alignment^30^. As the Nile tilapia reference genome is only in its second release (oreNil2) with many contiguous regions and gaps, our dataset mapping efficiency is the best we can achieve given available genetic toolkits today. Another possible reason for lower mapping efficiency was due to our samples being hybrids of *O. mossambicus* and *O. niloticus*, with genetic contributions from two closely related species, while the reference genome is from *O. niloticus*. Cytosine methylation level at single base-pair resolution was extracted using the software methylKit in R-environment^32^. Using the read.bismark function, a minimum reads coverage of 5X was set as the minimum parameter for calling CpG, CHH and CHG methylation states from sorted SAM file outputs generated by bismark^30^,^33^. At 5X coverage, an average of 47.9% of all CpG in the genome were covered by the sequencing data. An average of 20,334,256 cytosine methylation positions (combination of CpG, CHG and CHH at both Watson and Crick strands) were called for each sample. Based on our mapping results, on average 69.60% of cytosines in CpG context are methylated. In CHH and CHG context, only 0.57% and 0.47% of the cytosines are methylated respectively as summarized in the Supplementary Table 2. This result is consistent with the findings in vertebrates (e.g. *Tetraodon nigroviridis*)^34^, but is different from that in plants (e.g. *Oryza sativa*)^34^ (Fig. 1). CHH and CHG methylation is a major characteristic of plant methylomes and is largely absent or found in very low quantity in animal methylomes. In pufferfish, 65.50% of cytosines in CpG are methylated while cytosines in CHG and CHH are only methylated at 0.25% and 0.34%, respectively^34^. Other animal species showed similar traits lacking methylation on CHH and CHG cytosines as shown in Fig. 1. In the rice methylome, only 50.00% of CpG is methylated while 27.40% and 5.20% of cytosines are methylated in CHG and CHH context. These results suggest that cytosine methylation patterns are largely conserved in vertebrates, but are different in plant species.

**Figure 2:**
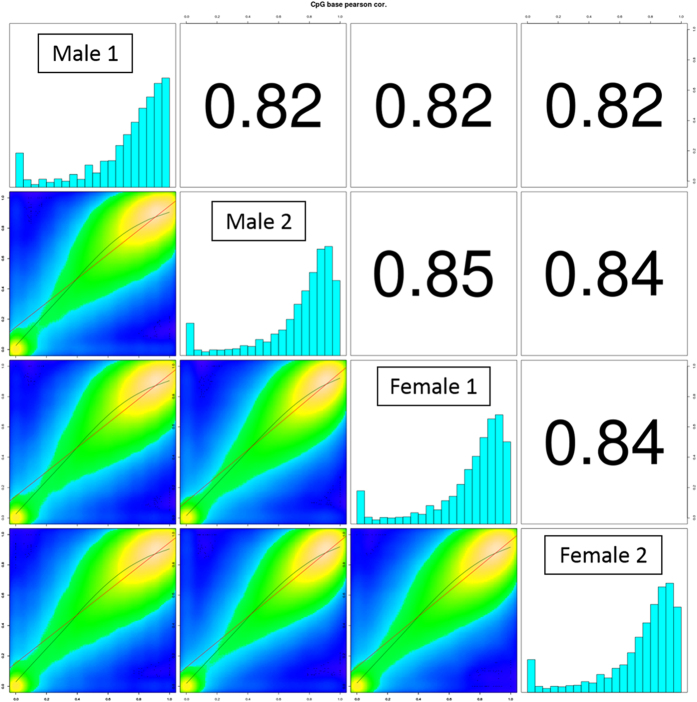
Correlation matrix showing the Pearson correlation of base resolution CpG methylation genome-wide between the skeletal muscle tissue samples of hybrid tilapia. Histograms showed CpG methylation level of each sample from 0% to 100% distributed across 20 bins of 5% intervals. The red line and green line represent linear regression and loess fit, respectively, to model the relationship of differential CpG methylation sites between compared individual pairs. Comparing the genome-wide CpG methylation profile between the sexes showed similar CpG methylation profiles in skeletal muscle tissue, with obvious differences between the sexes. Also, majority of CpG sites are highly methylated, with most of the CpG sites at more than 75% methylation level.

In Fig. 2, we present a comparison of genome-wide CpG methylation levels between the four samples studied in this report. Overall, the samples showed similarities of CpG methylation profile on a genome-wide scale. However, there are distinct regions of sexually dimorphic DMRs found in our analysis, which will be discussed in subsequent paragraphs.

### Tilapia gene body methylation patterns

**Figure 3:**
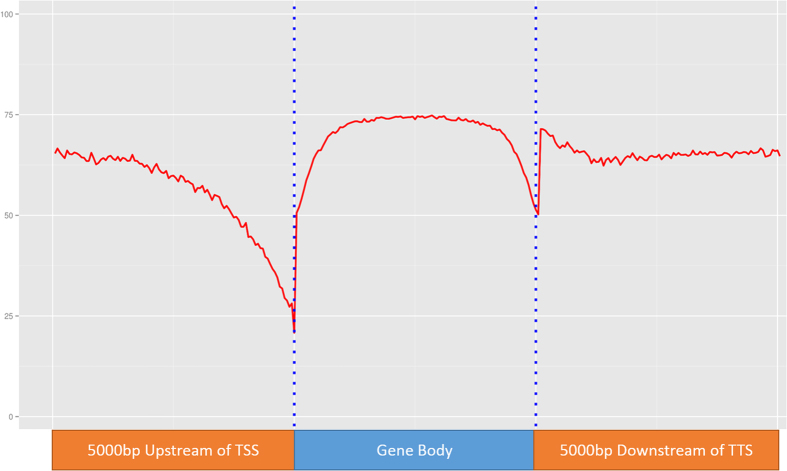
CpG Methylation level in relation to gene body, 5 Kb region upstream of TSS and 5 Kb downstream of TTS in hybrid tilapia. CpG methylation level dropped gradually to 25% approaching the TSS before rising sharply to 75% in the gene body. CpG methylation level then dipped rapidly to 50% approaching the TTS and subsequently returned to earlier intragenic CpG methylation level.

To investigate the methylation patterns of tilapia around the gene structure, we extracted the Trascriptional Start Sites (TSS), Transcriptional Termination Sites (TTS) and GeneScan-predicted mRNA coordinates from UCSC Genome Browsers. We then combined all four libraries and proceed to generate one high coverage methylome (>40X coverage) using the same procedure described above. We extracted the coordinates of 5 Kb upstream and 5 Kb downstream of the TSS and TES respectively and divided them into 100 bins. The average CpG methylation values for each bin was then called using SeqMonk (http://www.bioinformatics.babraham.ac.uk/projects/seqmonk/). mRNA tracks of 28,730 genes were downloaded from UCSC Nile tilapia GeneScan tracks and divided into 100 bins each. The mean methylation of each bin was calculated by averaging the methylation level of every CpG cytosine within the range. A gene structure methylation profile plotted with these data using ggplot2 in the R-environment^35^ is shown in Fig. 3. The CpG methylation level of tilapia on average drops to 23.00% approaching the TSS. Along the gene bodies, CpG methylation increased rapidly after TSS and reached a plateau of around 75.00% before dropping to 50.00% approaching the TTS. Between the TTS and 5 Kb downstream of TTS, CpG methylation levels averaged at 70.00%. The gene structure CpG methylation profile is consistent with other sequenced vertebrate methylomes^34^,^36^, which highlights the rapid drops in CpG methylation level approaching the TSS, which is a region highly enriched with transcription factor binding sites. Similar results were reported in plants^34^,^36^, fish^37^, and humans^38^, suggesting that the CpG methylation profiles of vertebrates on the gene structure are largely conserved from fish to humans. Our results are consistent with the observations by Suzuki *et al*.^4^, who observed global CpG methylation except on CpG islands, in contrast to the mosaic CpG methylation found in some plants (e.g. *Arabidopsis thaliana*), invertebrates (e.g. *C. intestinalis*) and fungi (e.g. *Neurospora crassa*). High CpG methylation within the gene body may be involved in a proposed mechanism to prevent initiation on the gene body instead of TSS to prevent the production of aberrant mRNAs^39^.

### Methylation profiles of various repeat elements in tilapia reference genome

**Figure 4:**
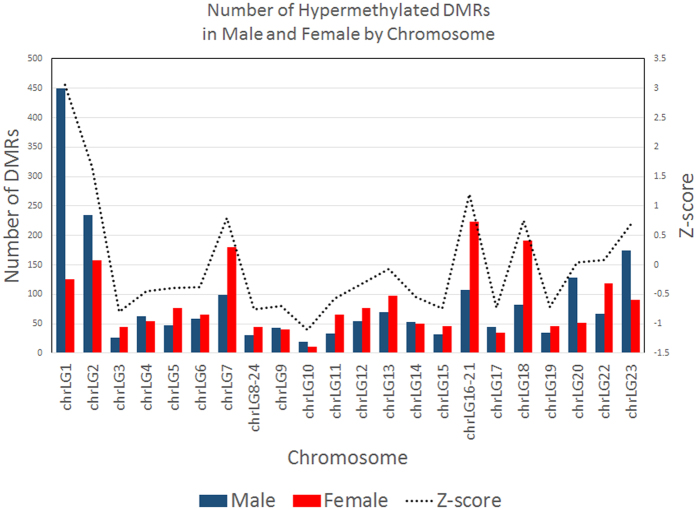
Distribution of CpG methylation level across various genomic elements such as promoters, introns, exons, DNA transposons, SINEs, LINEs and LTRs in male and female tilapia. The percentage of repeat elements is summarized according to chromosome locations.

We annotated the tilapia reference genome for repeat elements using RepeatMasker’s latest repeat library RepBase (26-5-2016)^40^. The parameters used were: **-s –gff –species vertebrates**. We selected **–s** (slow option) as it is up to 5% more sensitive but three times slower than the default parameter as described by RepeatMasker’s authors^40^. We also directed the RepeatMasker search mechanisms to target only repeats found in vertebrates, to reduce the numbers of false positives. In this annotation, we masked approximately 93.9 Mb (10.13% of the entire genome) as repeat elements. The annotation summary is shown in Supplementary Table 3. Up to 4.78% of the genome was found to be retroelements such as short interspersed nuclear elements (SINEs), long interspersed nuclear elements (LINEs) and long terminal repeats (LTRs). We also annotated 3.58% of the reference genome as DNA transposons. 1.35% of the genome was found to be simple repeats. We then calculated the total length of transposable elements in each chromosome divided by the length of the chromosome. We found no chromosome with outlying proportion of TEs, as shown by Fig. 4.

We profiled the CpG methylation levels of various genomic elements as high (>75% CpG methylation), medium (75–25% CpG methylation) and low (<25% CpG methylation) for both male and female skeletal muscle tissue, which is shown in Fig. 4. We found that in both sexes, more than 50% of DNA elements such as exons, introns, DNA transposons, LINEs, SINEs, LTRs and satellites were highly methylated (>75% CpG methylation). In promoter regions, close to 40% of the CpG sites were lowly methylated (<25% CpG methylation) while 25% of the promoter regions were highly methylated in both sexes.

### Sexually dimorphic differentially methylated regions revealed by high coverage whole genome bisulphite sequencing

**Figure 5:**
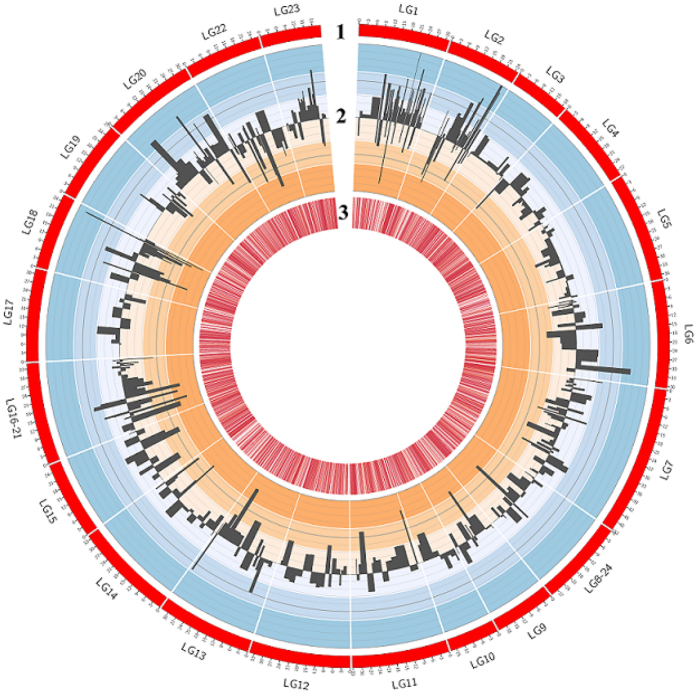
CIRCOS chart summarizing the differentially methylated regions in two sexes of hybrid tilapia. **Track 1** shows an ideogram representing the 22 chromosomes in hybrid tilapia. **Track 2** represents differentially methylated regions between the male and female individuals. Histograms in the blue regions show regions hypermethylated in males while orange regions show regions hypermethylated in females. Histograms were expanded for illustration purposes and histogram width is not a direct representation of DMR size. **Track 3** displays the locations of all CpG islands in hybrid tilapia.

**Figure 6:**
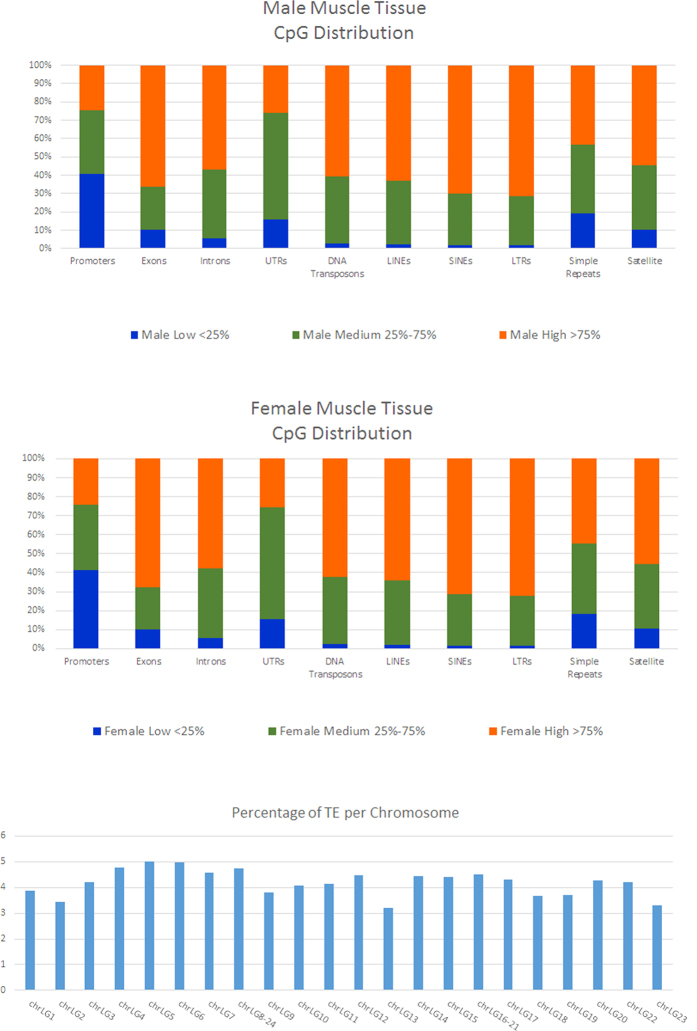
Numbers of hypermethylated DMRs in male (blue) and female (red) tilapia skeletal muscle tissue. Dotted line graph represents the Z-score of number of DMRs in each chromosome. In this plot, chromosome LG1 shows very high number of DMRs as compared to the other chromosomes (Z-score > 3).

Base-pair resolution of methylome was extracted from bismark SAM files using the R-package methylKit^32^. We imposed a minimum requirement of 5X coverage per cytosine in order to call on the CpG methylation percentage for each CpG position, as our data shows that at 5X coverage, on average 47.9% of all CpG sites were covered with minimally 5 reads. Samples were divided into biological replicate sets of male and female with two replicates each. PCR replicates were removed by filtering out the top 0.10% CpG with the highest sequencing coverage. While the CpG methylation profiles are largely similar between the samples, we found regions of significant DMRs between the male and female tilapia at both base and region levels. For CpG base-level differential methylation analysis, 361,702 genome-wide CpG coordinates with a minimum 5X coverage in every sample were extracted for differential methylation calculation. A total of 17,112 CpG sites were found to be differentially methylated between male and female tilapia muscle tissue with a q-value (FDR corrected p-value) cut-off point of 0.01 and methylation differences of 25%. However, single base-pair methylation is often not informative enough to infer functions for DMRs on a genomic scale, partly due to missing data points which gave heavy penalties to the q-values when calculating differential methylation. A sliding window analysis is more appropriate as it represents regions of differentially methylated cytosines instead of single cytosines^32^. Also, the methylation level of each window can be averaged across the window frame, providing compensation for lost single base-pair data points or CpG sites with low coverage in some libraries. We performed a sliding window analysis with a window size of 1000 bp at a stepping size of 1000 bp^32^. Using the **calculateDiffMeth()** function in methylKit^32^, a total of 4,757 windows were identified as DMRs with a q-value (FDR corrected p-value) cut-off point of 0.01 and minimal CpG methylation difference of 25%. We defined DMRs as 1000 bp windows with minimally 25% CpG methylation difference between two groups with a FDR corrected p-value of 0.01. Out of these 4,757 DMRs, 2,360 DMRs were hypermethylated in female skeletal muscles while 2,397 DMRs were hypermethylated in male skeletal muscles. The DMRs have a combined size of 4.76 Mb, representing 0.45% of the genome assuming the genome size of tilapia is 1060 Mb. The list of DMRs is provided in Supplementary Table 4. The CpG methylation landscape in the two sexes of tilapia is summarized in Fig. 5 in CIRCOS format, shown with the density of CpG islands in each chromosome. The DNA methylation patterns in different chromosomes were different between the two sexes as shown in Fig. 6. We summarize the numbers of DMRs with more than 25% difference in hypermethylated CpG levels according to their respective chromosomes in Fig. 6. In this graph, we can clearly see that in LG1, we observed a high number of DMRs located on LG1 (449 hypermethylated DMRs in male and 125 hypermethylated DMRs in female) with a Z-score of more than 3, indicating an outlier. We also observed moderately high numbers of DMRs located on LG2, LG7, LG16-21, LG18 and LG23 as summarized in Fig. 6. Some (e.g. LG23) of these linkage groups were previously shown to be associated with sex determinations. Genetic markers linked to sex determination were mapped to regions in chromosomes LG1 and LG23^24^,^25^,^41^. An XY sex determining system was identified on LG1 in *O. niloticus*^24^. In *O. aureus*, epistatic interactions between a WZ system on LG3 and the initial XY system on LG1 were identified^37^. Two other distinct quantitative trait loci (QTL) for sex determination were reported on LG23 in a hybrid cross between *O. aureus* and *O. mossambicus*^25^.

To understand the biological significance of the DMRs hypermethylated in male and female tilapia, we scanned for overlapping regions of DMRs with arbitrary promoters of 1000 bp upstream of each gene. We found 89 hypermethylated promoters in male tilapia and 60 hypermethylated promoters in female tilapia. We summarise these findings in Supplementary Table 5 to show the name of the hypermethylated gene and the expression level of each gene in log2 RPM in all libraries.

Our results suggest that sexual dimorphic patterns of genome-wide CpG methylation are present between male and female skeletal muscle tissue, especially in chromosomal regions where QTL for sex-determination were mapped. An epigenetic regulatory mechanism may be in place to regulate the expression of genes in the muscle tissue, which leads to the phenomenon of sexual size dimorphism in tilapia. However, whether sex determines the patterns of DNA methylation or vice versa remains unknown, as our results can only show association of specific DMRs with each sex. The biological significance of these DMRs will be the target of future research.

### Transcriptome analysis

**Figure 7:**
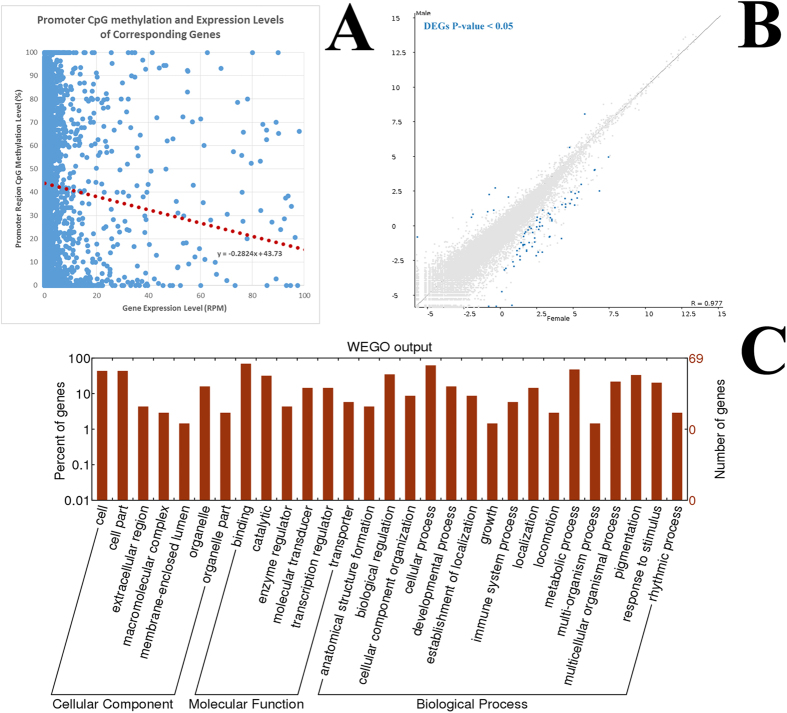
Low CpG methylation at promoter regions are linked with high gene expressions in skeletal muscle of hybrid tilapia. (**A**) Log_2_ RPM vs CpG methylation levels (%) at gene promoter regions. Gene promoter regions are arbitrary defined as 1000 bp upstream of TSSs. (**B**) Averaged log_2_ (RPM) profile of male (Y-axis) and female (X-axis). Differentially expressed genes (DEGs) are highlighted in green with multiple testing corrections. (**C**) WEGO output of sexually dimorphic DEGs.

A total of 76,452,907 read pairs were obtained, encompassing about 32.9 Gb of sequencing data. All four libraries were mapped successfully to the Nile tilapia Ensembl genome using the STAR RNA-seq Aligner with mapping efficiency at 91.67%, 90.99%, 92.62% and 92.55% for M1, M2, F1 and F2 libraries respectively^42^. The sequencing statistics summary is shown in Supplementary Table 1. A slight bias of mapping efficiency (~2% more) for the female libraries is because the Nile tilapia reference genome was generated from a female individual derived from an inbred line from Stirling University^19^. The combined total number of reads is 71,628,567 read pairs, averaging 17,907,141 read pairs (2 × 100 bp) per library. The reads were annotated and quantified using the mRNA tracks from Ensembl, which comprised of 28,730 transcript tracks, and using the software SeqMonk (http://www.bioinformatics.babraham.ac.uk/projects/seqmonk/). To prevent redundancies in transcript abundance reporting, only unique tracks were reported. M1 and M2 were regarded as biological repeats for the Male group while F1 and F2 were regarded as biological repeats for the Female group. All four libraries were quantified in log_2_ RPM (reads per feature per million reads of library) and in raw read counts. Log_2_ RPM quantifications were used for relative abundance estimations. Raw read counts were called for DEseq2 DEG statistics calculation. Using DEseq2, DEGs were calculated for Male and Female groups with multiple testing corrections applied and a cut-off FDR-corrected P-value of 0.05. A total of 78 DEGs between males and females were found in the muscle tissue with a false discovery rate (FDR) lower than 0.05. The DEGs were listed in Supplementary Table 6. Figure 7A shows the scatterplot of mean log_2_ RPM values for Male against Female libraries, with the DEGs (q-value < 0.05) highlighted.

The Gene Ontology (GO) numbers for these DEGs were extracted using Ensembl BioMart tools. GO provides a generalised description of the transcript products in terms of the molecular functions, biological processes and the cellular components of which the transcript of interest is associated with. The extracted GO list for the DEGs were entered into WEGO for GO classification studies^43^. Figure 7C displays the functional classifications of tilapia transcripts within the major classifications of cellular component, molecular function and biological processes. In terms of biological functions, most of the transcripts fall in the categories of biological regulation, cellular process, developmental process and metabolic processes. These gene groups may explain the sexual size dimorphism of tilapia as some of the genes listed amongst the DEGs (Supplementary Table 6) are lipid metabolism-related, such as *Lipin*-1 and *Lipin*-2, which are two well-studied genes that are linked to obesity^38^. Our results showed that only a small number of genes are classified under the growth category (e.g. epidermal growth factor receptor a) while a majority of these DEGs are related to regulation of transcription activity (e.g. estrogen-related receptor alpha & cAMP responsive element binding protein 5a), suggesting that sexual size dimorphism may have an upstream affecter mechanism rather than downstream effector mechanism such as those that are growth related. Also, other tissues such as liver and pituitary glands may have contributed more towards SSDs than skeletal muscle tissues.

Among the DEGs detected (Supplementary Table 6), 10 genes were highly expressed in males, while 68 were highly expressed in females. Of the 10 DEGs in males, we found 2 genes that are central to the MAPK/ERK signalling pathways, namely the G-protein subunit-5-beta (ENSONIT00000003305) and Ras protein-specific guanine nucleotide-releasing factor (ENSONIT00000019125). The MAPK/ERK signalling pathway is involved in regulating various transcription machineries via receptor interactions with various growth factors from extracellular domains^44^. The higher expression of genes in this pathway in males suggests that this pathway may be crucial in sexual size dimorphism in hybrid tilapia.

**Figure 8:**
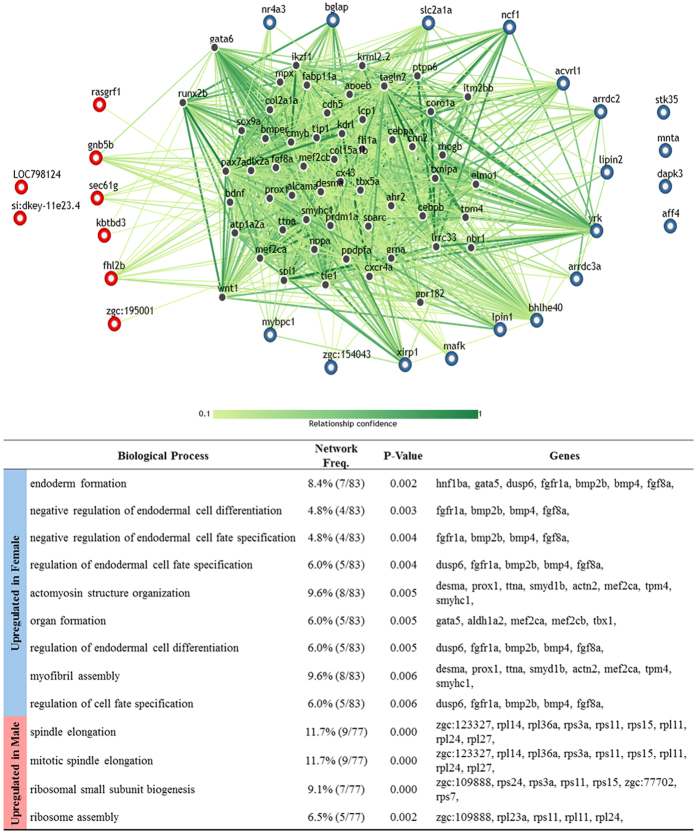
Gene network analysis of DEGs. Genes upregulated in males are designated as red nodes while genes upregulated in females are designated as blue nodes. Relationship confidence level is depicted in terms of green colour tones. The gene networks involved are listed in the table with the corresponding biological process associated with the networks shown. P-values shown are FDR corrected.

We also looked into the genetic network involving the DEGs discovered in our experiment. Using the web application IMP 2.0 (http://imp.princeton.edu/), we input the zebrafish genes analogous to our DEGs to create a predicted gene network model based on the gene network modelled in zebrafish^45^. The resulting network map is shown in Fig. 8. We applied our experimental data to the gene network database and found that the majority of the genes overexpressed in females were related to endoderm formation (8.4%), regulation of endodermal cell fate specification (6.0%), actomyosin structure organization (9.6%) and regulation of cell fate specification (6.0%). However, the genes with elevated expression in males were found to be part of the genetic networks involved in mitotic spindle elongation (11.7%), ribosomal small subunit biogenesis (9.1%) and ribosome assembly (6.5%), suggesting that the tissues found in males have fixed cell fates and are progressing with somatic growth.

### CpG methylation in promoters is negatively correlated with the gene expression

We extracted the CpG methylation level for an arbitrary promoter region 1000 bp upstream from TSS for each gene and compared the methylation level to the respective mRNA expression level. We found a general trend whereby low level of methylated CpG 1000 bp upstream from TSS was associated with increased expression level of the corresponding gene as shown in Fig. 7A. In other words, an enrichment of methylated cytosine leads to low expression level on the corresponding gene. A scatterplot of the CpG methylation level in the arbitrary promoter region (1000 bp upstream of TS) against log_2_ RPM for each corresponding gene is shown in Fig. 7. A linear regression trend line (red) shows the relationship between promoter CpG methylation level and gene expression level. Gene suppression effect is most robust when the CpG methylation level in the corresponding promoter region is high. This finding is consistent with reported vertebrate methylomes such as human peripheral blood mononuclear cells (PBMCs), mice and tetraodon fish^46^. Our data extends the conventional view that promoter CpG methylation in teleost genomes can suppress gene expression, similar to the observations found in mammalian genomes. As such, the mechanism of suppressing gene expression via CpG methylation in the promoter regions may have an even earlier evolutionary origin than teleosts.

### Whole genome bisulfite sequencing reveals sexually dimorphic regulatory landscape

**Figure 9:**
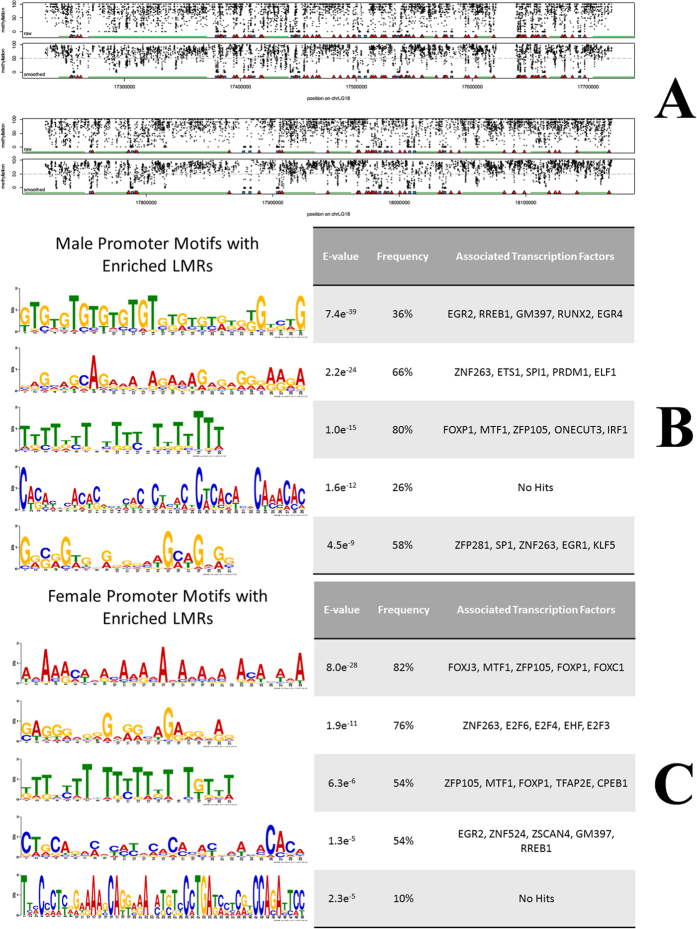
Enrichments of sexually dimorphic active cis-regulatory motifs in hybrid tilapia skeletal muscles. Only the top 50 genes in each sex with the highest enrichments of LMRs in the promoter regions were selected for analyses. Transcription factor motif binding sites were drawn from the database JASPAR. (**A**) Male coordinates of LMRs (red triangle) and UMRs (blue square) in chromosome LG18. (**B**) Consensus sequence motifs of promoters enriched with LMRs in males. (**C**) Consensus sequence motifs of promoters enriched with LMRs in females.

Encouraged by the development of robust algorithms to interpret high coverage WGBS data^15^,^47^, we intended to explore the regulatory elements of hybrid tilapia with the aim of identifying non-coding regulatory elements that may provide evidence to explain the phenomenon of sexual size dimorphism. Using the WGBS data generated for tilapia, we inferred the ACRs from the hybrid tilapia genome using the software MethylSeekR. We generated a Biostrings-based R library, BSgenome.Oniloticus.UCSC.orenil2, as the reference genome for MethylSeekR analysis. A methylation cutoff point of 50% and a false discovery rate threshold of 0.05% were set as parameters for segmentation of genomic regions into low methylated regions (LMRs) and unmethylated regions (UMRs), as recommended by the authors of MethylSeekR^15^. Partially methylated regions (PMDs) were identified and masked. PMDs were defined as contiguous regions with an average methylation level less than 70%. These PMDs are characterized by highly disordered methylation, resulting in an average methylation clearly below the genomic background level. It is essential to mask these PMDs to reduce false positive hits^15^. From a previous study done in mice, LMRs were found to be associated with active occupancy by DNA-binding factors^14^. Using the algorithms in MethylSeekR, we intended to infer the ACRs in the tilapia genome with the aim to uncover regions of transcription factor occupancies. From the WGBS datasets, we inferred 101,476 LMRs and 16,154 UMRs from the female methylome (Supplementary Table 7) while 83,697 LMRs and 16,318 UMRs were inferred from the male methylome (Supplementary Table 8). We then defined arbitrary promoter regions as 500 bp upstream of transcriptional start sites and performed enrichment quantifications for LMRs that are present within these arbitrary promoter regions. We defined the promoter as 500 bp upstream of TSS in this section of our analyses as we are interested in transcription factor binding sites that are located close to the TATA-box which is usually located 25–35 base pairs upstream of the transcription start site. Defining a promoter region that is too long (e.g. 1000 bp) in this analyses may lead to false positives. The top 50 promoter regions for each sex with the highest enrichment for LMRs were then selected to extract the FASTA sequence and were analyzed using the MEME suite for motif analyses^48^. We selected five common motifs returned with the lowest E-value and proceeded to search for their corresponding transcription factors from the JASPAR database^49^. The resulting motifs and the associated transcription factors are listed in Fig. 9. Most of these transcription factors are responsible for the development of skeletal and heart muscle (RUNX2, FOXP1, MTF1, and FOXJ3) and the development of the nervous system (EGR2, EGR4, FOXC1 and TFAP2E). We also found increased enrichment of transcription factor binding site motifs that are associated with the immune system in the male group (ETS1, SPI1, PRDM1, ELF1 and IRF1). In the female group, we found an enrichment of motifs related to the cell cycle (E2F3, E2F4, and E2F6), development (EGR2b, TFAP2e and FOXC1a) and negative regulation of epithelial cells (EHF). Previous studies have shown that transcription factors bound to cis-regulatory elements (CRE) found on the promoter regions upstream of TSSs will shape the CpG methylation levels on the CRE^47^. It is conventionally understood that highly methylated promoter regions are associated with suppressed gene expression and vice versa. Gene bodies, on the other hand, are often highly methylated when expression level is high^5^.

Using WGBS data to infer and predict ACRs adds value to the WGBS datasets, which are often criticized as expensive and inefficient, as only CpG methylation base pairs from WGBS were used for analysis. By complementing WGBS with ChIP-seq, a more comprehensive cis-regulatory landscape can be inferred as it would include epigenetic regulatory regions that were inferred from regions with histone modifications. In *O. niloticus*, a ChIP-seq dataset derived from anal fin tissue^50^ is available. Our data will complement the existing available data for regulatory region analysis. To functionally analyse cis-regulatory regions, candidate regions mapped can be analysed with STARR-seq for quantitative analysis of enhancer regions^51^. Due to higher interest in non-coding regions of DNA and their role in evolution, polymorphisms in CREs are thought to be responsible for causing adaptive phenotypic complexity through changes in gene expression and cell identity development^14^,^50^,^52^. CREs are important genetic tools for in-depth analysis on the evolution of vertebrate transcriptional regulation. Although mutations in coding sequences that result in amino acid changes are often responsible for many phenotypic divergences, they are often expected to be pleiotropic, affecting more phenotypes and are more likely to be deleterious due to dis-functional amino acids. Phenotypic changes in CREs on the other hand, are thought to be more modular and tissue specific, as any changes in CREs will only impact cells in particular tissues affected by the expression changes due to CREs^14^. With improved knowledge on the coordinates of regulatory elements, more in-depth analysis can be done on genetic variants found on these non-coding regulatory elements, using data generated from QTL and GWAS experiments. Possible changes in expression due to polymorphisms in CREs may explain the phenotypic variation occurring due to differential expression.

In summary, we presented the tilapia DNA methylome at base resolution, highlighting the differences between male and female tilapia methylomes. We found extensive differences between male and female tilapia methylomes, revealing the dynamic nature of CpG methylation in sexually dimorphic manners. We showed that the majority of cytosines in CpG context were largely methylated and CpG methylation in the promoter regions of tilapia were associated with suppressed gene expression. We also quantified the CpG methylation level in repeat elements in the tilapia genome, showing that most of the repeat elements, such as DNA transposons, LINEs, SINEs and LTRs, were highly methylated. We mapped UMRs and LMRs on the Nile tilapia reference genome (oreNil2), revealing a sexually dimorphic cis-regulatory landscape in the tilapia genome. We highlighted a sexually dimorphic regulatory landscape in tilapia, which may be involved in sexual dimorphism in tilapia. We inferred different active transcriptional factors on CREs in male and female tissues. This study will allow future works to quantitatively characterise evolutionary important CREs via other protocols such as STARR-seq^51^.

## Methods

### Ethical statement

All experiments in this study were approved by the IACUC Committee of Temasek Life Sciences Laboratory (Approval number: TLL(F)-11-001), and the experiments were performed according to the regulations and guidelines established by this committee.

### Sample collection

Tilapias were cultured to 108 days post hatch (dph) at ambient temperature in the marine fish culture facility in Temasek Life Sciences Laboratory. The tilapias were derived from a F_2_ hybrid strain (*O. niloticus* X *O. mossambicus*) bred for growth out in a full seawater (30–34 ppt). Fish were fed twice daily with commercial feed (BioMar, Aarhus, Denmark) and maintained in a recirculating tank. The water temperature was 25–28 °C during the experiments. At 108 dph, two male and two females were selected based on genital organ external appearances. Body mass, total length and standard length of the individuals were measured (Supplementary Table 9). Fishes were anesthetized with AQUI-S (AQUI-S, Lower Hutt, New Zealand) at 17 mg/L concentration for 3 minutes and sampled for downstream analysis. Skeletal muscle tissues were sampled immediately and excised into small pieces and divided into two portions, one stored in TriZOL (Thermo Fisher Scientific, Waltham, USA) solution for RNA extraction while the other portion was stored in pure ethanol for DNA extraction. All tissues were stored in a −80 °C freezer until RNA/DNA extraction.

### Genomic DNA extraction from skeletal muscle tissues

Genomic DNA was extracted from tilapia skeletal muscle tissues. Briefly, excess ethanol was removed. 50 mg of dried sample was then lysed in 300 μl SET buffer (0.4 M NaCl, 10 mM Tris-HCl pH 8.0, 2 mM EDTA pH 8.0 and 2% SDS) with 20 μg of proteinase K (Roche Life Sciences, Basel, Switzerland) in an orbital shaker at 250 revolutions per minute (rpm) with temperature set to 55 °C for 90 minutes. 0.5 mg of RNAse A (Qiagen, Hilden, Germany) was added into the lysate and incubated for 15 minutes at room temperature. 400 μl of 5 M NaCl was added into the sample mixture and vortexed briefly until homogenous. The mixture was then centrifuged at 4 °C at 13,000 rpm for 30 minutes. The supernatant (approx. 600 μl) was transferred to a new Eppendorf tube and 600 μl of isopropanol was added and mixed briefly. The DNA-isopropanol mixture was precipitated overnight in a −20 °C freezer and then centrifuged at 4 °C at 13,000 rpm for 30 minutes. The resulting DNA pellet was then washed twice with 80% ethanol and dissolved in Tris-EDTA pH 8.0 buffer and stored at −80 °C until ready for bisulphite conversion.

### Whole genome bisulphite sequencing and data processing

Two μg of DNA samples were spiked with one ng of unmethylated lambda DNA (Promega, Fitchburg, USA) as internal control for bisulphite conversion efficiency. The DNA mixture was fragmented to 100–300 bp by sonication using Covaris M220, (Covaris Inc, Woburn, USA) followed by DNA-end repair, dA addition at 3′ end and ligation of sequencing adaptors and index. The resulting mixtures were used for bisulphite conversion on the ZYMO EZ-DNA Methylation-Gold kit (Zymo Research, Irvine, USA) following standard protocol. Size selection was then conducted on the Pippin-Prep platform (Sage Science, Beverly, USA) with target size in the range of 300–320 bp. The final library was then on Illumina HiSeq 2000 (Illumina, San Diego, USA) and sequenced with 2 × 100 bp paired-end protocol by BGI-Shenzhen (BGI-Shenzhen, Shenzhen, China). Raw reads were converted to FASTQ format and demultiplexed using bcl2fastq V 2.16 (Illumina, San Diego, USA). The resulting FASTQ reads were then quality controlled using the IlluQC.pl module from NGSToolKit Version 2.3^29^.

The clean reads were aligned to the reference genome oreNil2^19^ using the suite of software in Bismark v0.14.4^30^. The reference genome of Nile tilapia, oreNil2, was downloaded from the UCSC Genome Browser website^19^. The two strands of oreNil2 were modified in silico to convert all C’s to T’s, using the bismark_genome_preparation software with indexing format following Bowtie2 requirements^30^. Reads were mapped to these two modified genomes using bismark with the following parameters: **-q –p –N 1 –bowtie2**. The resulting BAM files were sorted and converted to SAM format using Samtools V1.4^53^. Subsequently, differentially methylated region analysis was conducted using Methylkit in the R-environment^32^. The parameters for **calculateDiffMeth()** in methylKit were as follows: **slim** = **TRUE, weighted.mean** = **TRUE, num.cores** = **12**.

RepeatMasker was used to annotate the repeats elements found in the tilapia reference genome^40^. The parameters used for RepeatMasker were as follows: -pa 5 –s –gff –species vertebrates. The generated GFF files were then used to annotate the tilapia genome for calculation of methylation profiles across various repeat elements.

MethylSeekR was used to infer regulatory element landscape in the R-environment^15^. Biostrings-based reference genome data were required for MethylSeekR, but was unavailable for the Nile tilapia reference genome as it is not a model species. Hence, a Biostrings-based R library, ***BSgenome.Oniloticus.UCSC.oreNil2.tar.gz***, was created for oreNil2 using BSgenome in the R-environment^54^. Subsequently, UMRs and LMRs were identified using MethylSeekR with the Partially Methylated Domains (PMDs) masked. An arbitrary promoter region was defined as 500 bp upstream of the transcriptional start site for each transcript. UMRs and LMRs were mapped to their respective coordinates on SeqMonk (http://www.bioinformatics.babraham.ac.uk/projects/seqmonk/). All arbitrary promoter regions were then scored and quantitated based on an enrichment test for LMRs on SeqMonk. Motifs analyses were conducted on the MEME suite^48^.

### Transcriptome sequencing and data processing

Total RNA was extracted from tilapia skeletal muscle tissue using TRIzol (Thermo Fisher Science, Waltham, USA) following standard protocol. Poly-A tailed mRNA was enriched using oligo-dT for library preparation. The extracted mRNA was fragmented by Covaris M220 (Covaris Inc, Woburn, USA), using mRNA fragmentation standard factory default protocol, and reverse transcribed into cDNA. 5′ and 3′ Illumina adaptors and indexes were ligated onto the cDNA produced and size selected for 2 × 100 bp paired-end sequencing on the Illumina HiSeq 2000 (Illumina, San Diego, USA) using standard paired-end protocol settings at BGI-Shenzhen (BGI-Shenzhen, Shenzhen, China). Raw sequencing output was processed and de-multiplexed into FASTQ format for each individual via bcl2fastq using default parameters. Raw FASTQ reads were quality controlled using the IlluQC.pl module from NGSToolKit Version 2.3^29^. The retained reads were then mapped onto the Nile Tilapia reference genome oreNil1.1 (Ensembl) using STAR, an ultrafast universal RNA-seq aligner^42^. The resulting SAM file was loaded onto SeqMonk to compute the log_2_ RPM using the mRNA tracks from the Ensembl database and the datasets were normalised to remove any trace of bias from read coverage differences. Differentially expressed genes (DEGs) were calculated using DESeq2 in the R-environment^55^. The resulting DEGs were then extracted for Gene Ontology IDs and analysed on WEGO^43^. Gene network analysis was conducted using IMP 2.0 with the gene network curated from a zebrafish gene network database^45^. The parameter used was a maximum of 75 genes in the gene set network analysis.

## Additional Information

Accession codes: WGBS reads and RNA-seq reads used in this study have been deposited into NCBI BioProject with the ascension codes PRJNA309880. 

**How to cite this article**: Wan, Z. Y. *et al*. Genome-wide methylation analysis identified sexually dimorphic methylated regions in hybrid tilapia. *Sci. Rep.* **6**, 35903; doi: 10.1038/srep35903 (2016).

**Publisher’s note:** Springer Nature remains neutral with regard to jurisdictional claims in published maps and institutional affiliations.

## Supplementary Material

Supplementary Information

Supplementary Information

Supplementary Information

Supplementary Information

Supplementary Information

Supplementary Information

Supplementary Information

Supplementary Information

Supplementary Information
